# The Complexities of Orbital Arteriovenous Malformations: A Systematic Review of Clinical Features and Treatment Approaches

**DOI:** 10.7759/cureus.71323

**Published:** 2024-10-12

**Authors:** Injam Ibrahim Sulaiman, Mohammed A Hashim, Mustafa Ismail

**Affiliations:** 1 Department of Surgery, College of Medicine, Hawler Medical University, Erbil, IRQ; 2 Department of Surgery, College of Medicine, University of Baghdad, Baghdad, IRQ; 3 Department of Surgery, Medical City Complex, Baghdad Teaching Hospital, Baghdad, IRQ

**Keywords:** embolization, imaging modalities, orbital arteriovenous malformations, proptosis, surgical resection

## Abstract

The orbital vascular malformations are very rare congenital lesions characterized by a connection directly between arteries and veins, thus bypassing the capillary network. These lesions may be associated with clinical features ranging from mild proptosis to vision-threatening conditions like visual loss and elevated intraocular pressure. Despite advances in imaging and treatment strategies, the management of orbital arteriovenous malformations (AVMs) still remains daunting because of the high risk of complications, including hemorrhage and incomplete resection. This systematic review was done according to the Preferred Reporting Items for Systematic Reviews and Meta-Analyses (PRISMA) guidelines. A detailed search of the major databases such as PubMed and Scopus was performed using the keywords "Ocular OR orbit AND arteriovenous malformations OR AVM" without specifying the date. All case reports, case series, and observational studies reporting the diagnosis, treatment, and outcome of patients with orbital AVM were included. Data extraction was focused on clinical presentations, diagnostic modalities, treatment interventions, and outcomes; narrative synthesis was done due to heterogeneity in the studies. This review contains 27 case reports, where the number of patients ranged between three years and 75 years with a mean age of 34 years. The majority of the patients were males constituting 66%. The common clinical features include proptosis in 16 cases, visual loss in 12 cases, and chemosis in seven cases. Most of the patients underwent surgical resection and embolization of the malformations. In most of the cases, surgical resection was uneventful; however, a few cases with high risks showed hemorrhage and loss of vision as complications. Conservative management was also effective in certain stable or asymptomatic cases.

Orbital vascular malformations include complex diagnostic and therapeutic challenges due to the complex anatomy of the orbit and the high risk of serious complications. This systematic review aimed at presenting the clinical features, diagnostic challenges, and treatment outcomes of orbital arteriovenous malformations for a better management strategy. Further research is required in the refinement of treatment protocols, especially regarding high-flow lesions, and a critical look at improving long-term efficacy.

## Introduction and background

Orbital arteriovenous malformations (AVMs) are rare, congenital vascular anomalies that are characterized by abnormal communications between arteries and veins that bypass the normal capillary bed [1]. While congenital, these lesions may not appear until later in life. Trauma or hormonal changes, such as those experienced during puberty or pregnancy, are often triggering factors [1]. The anatomy of the orbital cavity is complex, and a predisposition toward space-occupying lesions makes diagnosis and management of such malformations quite challenging. The complications are in the form of visual impairment, proptosis, and increased intraocular pressure; hence, the complications need to be diagnosed early along with appropriate treatment modalities [2].

In AVMs, the pathophysiology includes the shunting of high flow between the arteries and veins. This usually results in progressive growth that eventually leads to serious morbidity [3]. Typically, most AVMs of the orbit are similar to a central nidus made of abnormal vessels that have been fed by numerous arteries, usually branches of the ophthalmic artery. Because of the absence of the capillary bed, there is direct arterial inflow and venous drainage, and the resultant vessel dilation, wall thickening, and rupture or hemorrhage take place. The vessels have much hemodynamic stress that can eventually lead to spontaneous bleeding and thrombosis apart from predisposing clinical symptoms such as proptosis, loss of vision, and pain [3]. The high-flow nature of these lesions also puts the patient at greater risk for complications secondary to surgical or other interventional procedures due to the common threat of uncontrolled hemorrhage. These factors increase the hemodynamic load on the malformed vessels, often presenting the first clinical signs as proptosis or a decrease in visual acuity [1]. Advanced imaging, such as angiography and MRI, is important for better delineation of the nidus and abnormal flow patterns that may be important for diagnosis and treatment. Genetic mechanisms underlying AVMs include mutations that disturb the endothelial cell function during embryonic life, though the exact molecular pathways still remain an active area of investigation [1]. Overexpression of VEGF has indeed been found in several vascular malformations and may suggest its influence on lesion growth and the creation of collaterals supporting AVM progression [1].

Management strategies for orbital AVMs are really heterogeneous, ranging from simple observation to more interventional procedures such as embolization and surgical resection, based on the size and location of the lesion and its impact on ocular function. Many cases, due to risks associated with hemorrhage in the perioperative period, require a multidisciplinary approach to achieve favorable outcomes [4]. This review tries to comprehensively analyze the clinical presentations, diagnostic modalities, and management outcomes of orbital AVMs by synthesizing the available literature together with available case studies.

## Review

Method

**Figure 1:**
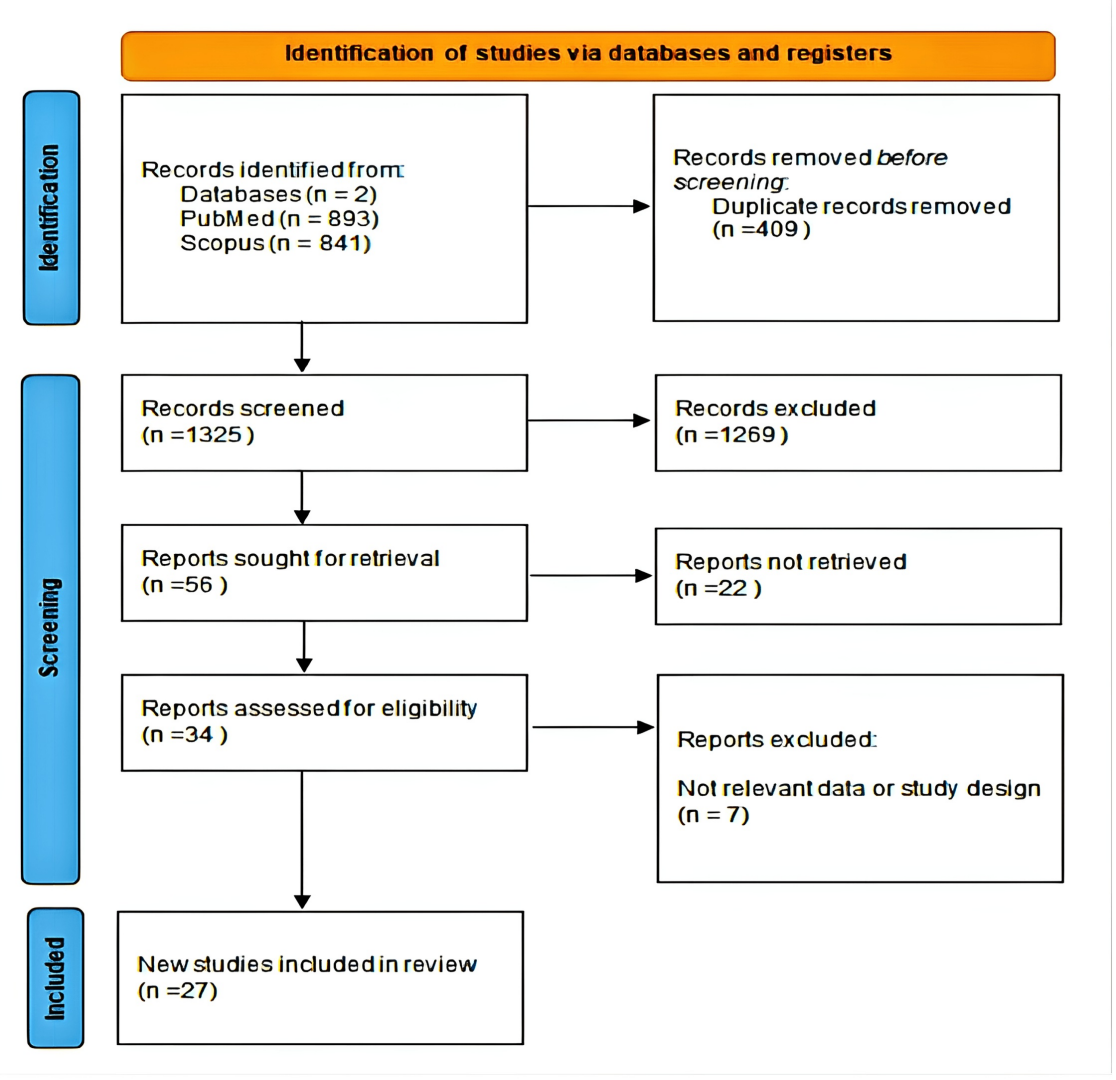
PRISMA flowchart of the included studies. PRISMA: Preferred Reporting Items for Systematic Reviews and Meta-Analyses

The Preferred Reporting Items for Systematic Reviews and Meta-Analyses (PRISMA) guidelines were followed to ensure that this systematic review was performed with methodological rigor and transparency (Figure 1) [5].

Search Strategy

For this review, a comprehensive literature search was done via several databases, including PubMed and Scopus. No date limits were put. The following search terms were used in different combinations: "Ocular OR orbit AND arteriovenous malformations OR AVM." The use of Boolean operators allowed for the wide retrieval of studies about orbital AVMs. No publication date limits were imposed on the papers to be retrieved. The complete search strategy can be found in the appendix. Initial screening of the large number of articles retrieved was done using the Rayyan tool (Doha, Qatar: Qatar Computing Research Institute). This platform facilitated blinded screening by multiple reviewers, ensuring that the selection process was free from bias. Duplicates were removed, and titles and abstracts were independently reviewed by two authors. Discrepancies in article selection were resolved by discussion, with a third author acting as an adjudicator when necessary.

Inclusion and Exclusion Criteria

The inclusion criteria for this review encompassed studies involving patients diagnosed with orbital arteriovenous malformations. The studies had to describe any diagnostic or therapeutic approach, including surgery, embolization, or conservative management, and report on relevant clinical outcomes such as vision preservation, resolution of proptosis, or complication rates. Eligible studies included case reports, case series, observational studies, and clinical trials. Conversely, reviews, editorials, and animal studies were excluded from the analysis.

Study Selection and Data Extraction

Data extraction was performed independently by two authors using a standardized data collection form. The following information was extracted from each study: author names, publication year, study design, sample size, patient demographics, clinical presentation, diagnostic modality, treatment interventions, and outcomes. All discrepancies were resolved through consensus.

Quality Assessment

The quality of case reports and case series was evaluated using the Case Report (CARE) Guidelines [6], ensuring that included studies met the necessary standards for completeness and transparency (Table 1) [7-33].

**Table 1:** Quality assessment of case reports on thalamic abscess using Case Report (CARE) Guidelines.

Study ID	Studies	Patient information	Clinical findings	Diagnostic assessment	Therapeutic interventions	Follow-up outcomes	Discussion/conclusions	Overall quality
1	Chakrabortty et al. (1993) [7]	Comprehensive	Detailed	Thorough	Well-documented	Reported	Relevant	High
2	Gil-Salú et al. (2004) [8]	Comprehensive	Detailed	Thorough	Well-documented	Reported	Relevant	High
3	Schatz et al. (1993) [9]	Comprehensive	Detailed	Thorough	Well-documented	Reported	Relevant	High
4	Schumacher and Wakhloo (1994) [10]	Comprehensive	Detailed	Thorough	Well-documented	Reported	Relevant	High
5	Hieu et al. (1997) [11]	Comprehensive	Detailed	Thorough	Well-documented	Reported	Relevant	High
6	Huna-Baron et al. (2000) [12]	Comprehensive	Detailed	Thorough	Well-documented	Reported	Relevant	High
7	Moin et al. (2000) [13]	Comprehensive	Detailed	Thorough	Well-documented	Reported	Relevant	High
8	Pathak-Ray et al. (2001) [14]	Comprehensive	Detailed	Thorough	Well-documented	Reported	Relevant	High
9	Lee et al. (2003) [15]	Comprehensive	Detailed	Thorough	Well-documented	Reported	Relevant	High
10	Shields et al. (2006) [16]	Comprehensive	Detailed	Thorough	Well-documented	Reported	Relevant	High
11	Trombly et al. (2006) [17]	Comprehensive	Detailed	Thorough	Well-documented	Reported	Relevant	High
12	Kaufman et al. (2007) [18]	Comprehensive	Detailed	Thorough	Well-documented	Reported	Relevant	High
13	Ho et al. (2010) [19]	Comprehensive	Detailed	Thorough	Well-documented	Reported	Relevant	High
14	Rouvas et al. (2011) [20]	Comprehensive	Detailed	Thorough	Well-documented	Reported	Relevant	High
15	Sato et al. (2011) [21]	Comprehensive	Detailed	Thorough	Well-documented	Reported	Relevant	High
16	Coca et al. (2013) [22]	Comprehensive	Detailed	Thorough	Well-documented	Reported	Relevant	High
17	Ahmed et al. (2014) [23]	Comprehensive	Detailed	Thorough	Well-documented	Reported	Relevant	High
18	Patel et al. (2017) [24]	Comprehensive	Detailed	Thorough	Well-documented	Reported	Relevant	High
19	Sinha et al. (2017) [25]	Comprehensive	Detailed	Thorough	Well-documented	Reported	Relevant	High
20	Tsui et al. (2019) [26]	Comprehensive	Detailed	Thorough	Well-documented	Reported	Relevant	High
21	Link et al. (2019) [27]	Comprehensive	Detailed	Thorough	Well-documented	Reported	Relevant	High
22	Gandhi et al. (2020) [28]	Comprehensive	Detailed	Thorough	Well-documented	Reported	Relevant	High
23	Accou et al. (2020) [29]	Comprehensive	Detailed	Thorough	Well-documented	Reported	Relevant	High
24	Rosenblatt et al. (2021) [30]	Comprehensive	Detailed	Thorough	Well-documented	Reported	Relevant	High
25	Dantas et al. (2021) [31]	Comprehensive	Detailed	Thorough	Well-documented	Reported	Relevant	High
26	Kamalden et al. (2008) [32]	Comprehensive	Detailed	Thorough	Well-documented	Reported	Relevant	High
27	Li et al. (2024) [33]	Comprehensive	Detailed	Thorough	Well-documented	Reported	Relevant	High

Data Synthesis

As only case reports and case series were identified, a quantitative synthesis or meta-analysis was not applicable. Instead, a comprehensive narrative synthesis was undertaken to collate and critically assess the clinical presentations, diagnostic approaches, management strategies, and outcomes reported in the selected studies. This synthesis aimed to identify common themes and insights that could inform future clinical practice in the management of orbital arteriovenous malformations.

Results

This systematic review included 27 case reports detailing the clinical manifestations, diagnostic findings, treatment approaches, and outcomes of patients with orbital AVMs (Table 2) [7-33]. The patients ranged from 3 to 75 years of age, with a mean age of 34 years across the studies. Most of the patients (66%) were males, while females comprised 34%. These patients presented with different ocular symptoms, the most common being proptosis, which was manifested in 16 cases, loss of vision in 12, and chemosis in seven. Other symptoms could have been related to ocular pain, diplopia, and ophthalmoplegia, depending on the size and position of the AVM.

**Table 2:** Summary of clinical presentations, diagnostic modalities, and treatment outcomes in orbital AVMs. AVMs: arteriovenous malformations; NBCA: N-butyl cyanoacrylate

ID	Studies	Sample size	Sex, N (%)	Age (mean, SD)	Study design	Location of study	Clinical manifestation	Location (orbital, retinal, ciliary body, optic nerve)	Size	Complication	Diagnostic modality	Imaging finding	Intervention type	Indication for surgery if applicable	Surgery approach if applicable	Complication from intervention	Outcome of intervention	Follow-up duration	Key findings
1	Chakrabortty et al. (1993) [7]	1	Male (100%)	27 years	Case Report	Kobe, Japan	Visual loss, exophthalmos, chemosis of the right eye	Orbital (retrobulbar, extraocular muscles, optic nerve involvement)	N/A	Complete vision loss in the right eye	CT, MRI, angiography	Enlargement of extraocular muscles, abnormal vascular stain with a dilated ophthalmic artery	Surgery (total removal of intraorbital contents)	Progressive symptoms, failure of conservative treatments	Transcranial extradural approach, orbitotomy	Cosmetic concerns post-surgery	Successful removal, unsatisfactory cosmetic outcome	N/A	Rare intraorbital AVM with optic nerve involvement, complex management due to anatomical variations
2	Gil-Salú et al. (2004) [8]	1	Male (100%)	18 years	Case Report	Castellón, Spain	Chemosis, bruit, without exophthalmos	Orbital (extrabulbar fatty tissue)	6 cm	None noted	CT, MRI, angiography	Vascular abnormality with extraorbital and intraorbital components	Surgery	Symptomatic with progression	Fronto-orbital craniotomy	None noted	Complete removal, confirmed by MRI and angiography	4 years	Successful surgical management of a large intraorbital AVM with no recurrence during follow-up
3	Schatz et al. (1993) [9]	2	Women (2, 100%)	Mean=34 years, SD=33.9	Case Report	San Francisco, USA	Visual loss, retinal hemorrhages, edema, central retinal vein occlusion	Retinal	N/A	Central retinal vein occlusion; neovascular glaucoma in case 2	Fluorescein angiography, clinical examination	Dilated, tortuous vessels; rapid filling of AVM; late staining	Conservative (observation)	N/A	N/A	N/A	Progression to neovascular glaucoma in case 2; sclerosis in AVM over time in case 1	Case 1: 8 years; case 2: 3 months	High flow and turbulence in AVM contribute to retinal vein occlusion; AVM can become sclerotic over time
4	Schumacher and Wakhloo (1994) [10]	1	Female (100%)	3 years	Case Report	Freiburg, Germany	Blindness, scarring, pulsating AVM over the right upper eyelid and supraorbital region	Orbital	N/A	Blindness due to prior radiotherapy	Angiography, CT	AVM fed by abnormal ophthalmic artery from the basilar artery	Embolization	Presence of AVM with cosmetic and functional impact	Not applicable (embolization performed)	None noted	Successful embolization, satisfactory cosmetic outcome, stable follow-up	N/A	Successful embolization of AVM in a complex case with an abnormal ophthalmic artery origin from the basilar artery
5	Hieu et al. (1997) [11]	1	Female (100%)	39 years	Case Report	Brest, France	Pulsating exophthalmos, visual loss, ocular pain	Orbital	N/A	None noted	MRI, angiography	Enlarged right ophthalmic artery feeding AVM, high blood flow	Surgery	Progressive symptoms, functional impairment	Fronto-orbital craniotomy with CO2 laser	None noted	Full recovery of vision, correction of proptosis, no recurrence on follow-up imaging	24 months	Surgery, including a fronto-orbital approach and CO2 laser, effectively managed intraorbital AVMs with functional restoration
6	Huna-Baron et al. (2000) [12]	2	Males (100%)	Mean=68.5 STD=3.5	Case Report	Tel Aviv, Israel; New York, USA	Eye pain, redness, proptosis, pulsatile sounds, chemosis, elevated intraocular pressure	Orbital	N/A	Visual impairment, severe chemosis, increased intraocular pressure	MRI, angiography	Small slow flow intraorbital shunt supplied by the ophthalmic artery, drainage to the superior ophthalmic vein and cavernous sinus	Transarterial and transvenous embolization	Poor response to conservative management	Transvenous embolization attempted, incomplete due to technical challenges	Incomplete occlusion of shunt, persistent symptoms	Partial improvement; no significant visual recovery	2 years	Orbital AVMs with slow flow can mimic cavernous sinus DAVM; treatment options are limited and often unsuccessful
7	Moin et al. (2000) [13]	1	Female (100%)	75	Case Report	Cincinnati, USA	Rapid onset of severe proptosis, chemosis, diplopia, painless	Orbital	2.5 x 1.8 x 2.0 cm	Intraoperative hemorrhage, severe visual loss, no light perception	CT, MRI, angiography	Intraconal mass with hemorrhage, honeycomb appearance on MRI	Surgery (orbitotomy, debulking), attempted embolization	Rapid progression of proptosis, visual loss	Lateral orbitotomy	Massive hemorrhage, no light perception in affected eye	Persistent proptosis, progression to enucleation	N/A	Orbital AVMs can cause spontaneous hemorrhage leading to severe complications; surgical management is challenging with high risk
8	Pathak-Ray et al. (2001) [14]	1	Female (100%)	7 years	Case Report	Sheffield, UK	Gross reduction of vision in the left eye, ipsilateral deficit in color vision, relative afferent pupillary defect	Retinal and orbital	N/A	Severe visual impairment in the affected eye	CT, MRI, ultrasound	Enlarged and tortuous retinal vessels with orbital involvement, no intracranial extension	Conservative (observation)	N/A	N/A	N/A	Stable condition with no progression over 3 years of follow-up	3 years	Rare presentation of ipsilateral retinal and orbital AVMs without systemic involvement, managed conservatively with stable long-term outcome
9	Lee et al. (2003) [15]	1	Male (100%)	33 years	Case Report	Singapore	Persistent dilated vessels in the nasal aspect of the left eye, redness, cosmetic concerns	Ciliary body	N/A	None noted	High-frequency ultrasound biomicroscopy (UBM)	Hypoechoic sheets and tubular structures in the ciliary body with communication between deep and superficial portions	Surgery (excision for cosmetic reasons)	Cosmetic concerns, patient elected for surgery	Cauterization and excision of dilated vessels with conjunctival autograft	None noted	No recurrence of dilated vessels, satisfactory cosmetic outcome	2 weeks post-op check showed stability	UBM is valuable in diagnosing ciliary body AVMs; surgical excision for cosmetic improvement can be successfully achieved without complications
10	Shields et al. (2006) [16]		9 male (64%), 5 female (36%)	Mean 49 years; median 50 years; range 16-79 years	Case Series	Philadelphia, USA; Bojnice and Zilina, Slovakia	Unilateral AVM of the iris, with vessels making loops (simple) or intertwining convolutions (complex); no significant systemic associations	Iris	N/A	None noted; no local or systemic complications	Slitlamp examination, fluorescein angiography	Uniformly hyperfluorescent lesions on angiography; minimal or no late leakage of dye	Conservative (observation)	N/A	N/A	None noted	No changes or complications during follow-up.	Mean 8 years; Median 7 years; Range None-14 years.	Iris AVMs are benign, stationary conditions with no systemic associations or complications; can be managed conservatively.
11	Trombly et al. (2006) [17]	14	Female (100%)	11 years	Case Report	Miami, USA	Vascular lesion involving the right upper and lower eyelids, and surrounding scalp; warm, palpable thrill, proptosis	Orbital	N/A	Persistent disabling swelling, necrosis of the right upper eyelid, cosmetic disfigurement	Magnetic resonance angiography (MRA), cerebral angiogram	Multiple arterial feeders, high-flow AVM; extensive feeding from ophthalmic, external carotid arteries	Serial embolization; attempted surgical resection	Progressive lesion size, cosmetic and functional concerns	Bicoronal incision, subtarsal lower lid incision; incomplete resection due to brisk bleeding	Persistent swelling, progressive necrosis, visual function deterioration	Persistent disfigurement; radical surgical resection planned, likely sacrificing vision in the right eye	Not specified	Complex, multidisciplinary approach needed; high-flow AVMs present significant management challenges, especially in pediatric cases
12	Kaufman et al. (2007) [18]	1	Male (100%)	12 years	Case Report	Houston, USA	Slowly progressive right proptosis, slight diplopia, limited upward gaze	Orbital	3.9 x 3.5 x 3.8 cm	None noted preoperatively; intraoperative bleeding noted during biopsy	CT, MRI, angiography	Soft tissue mass with aggressive lysis of the orbital roof; complex vascularity involving ophthalmic artery feeders	Surgery (orbitotomy, craniotomy); embolization planned but not executed due to technical challenges	Significant proptosis, risk of visual impairment, lesion enlargement	Bifrontal craniotomy, coronal incision	Mild residual proptosis	Resolved visual deficits and superior gaze limitation; residual cosmetic deformity	6 months	Intraorbital AVMs are rare and require complex surgical management; careful planning and multidisciplinary approach are essential for successful outcomes
13	Ho et al. 2010 [19]	1	Male (100%)	51 years	Case Report	Taipei, Taiwan	Redness of the left eye, proptosis, dilated conjunctival and episcleral vessels, intraocular pressure elevation, visual loss	Orbital	N/A	Neovascular glaucoma, central retinal vein occlusion	MRI, angiography, color Doppler sonography	AVM located near the orbital apex supplied by the ophthalmic artery and draining into the superior and inferior ophthalmic veins	Gamma Knife radiosurgery, transscleral cyclophotocoagulation	Progressive visual loss, uncontrolled intraocular pressure	Transscleral approach for cyclophotocoagulation	Persistent visual loss, progression to no light perception	Poor outcome with further visual deterioration	6 months	Orbital AVMs pose significant management challenges with limited treatment options; radiosurgery carries high risks and may not prevent severe visual loss
14	Rouvas et al. (2011) [20]	1	Female (100%)	27 years	Case Report	Athens, Greece	Extensive visual field loss, optic disc pallor	Peripapillary (retinal)	N/A	None noted	Fundus fluorescein angiography (FFA), indocyanine green angiography (ICGA), Doppler flow imaging	Focal dilatation and aneurysmal distention of the central retinal vein and large choroidal vein	Conservative management	N/A	N/A	N/A	Stability without further visual loss	Not specified	The case highlights the role of ICGA and FFA in assessing optic disc pallor and visual field loss; conservative management was chosen due to the congenital nature of the AVM
15	Sato et al. (2011) [21]	1	Male (100%)	53 years	Case Report	Sendai, Japan	Pulsating exophthalmos, chemosis, ocular pain, restricted eye movement	Orbital	N/A	None reported	Digital subtraction angiography (DSA)	AVM fed by branches from the left ophthalmic artery, draining into the facial vein	Transarterial embolization with N-butyl cyanoacrylate (NBCA)	Progressive symptoms, poor response to conservative management	Transarterial approach	None noted	Improvement in symptoms, complete occlusion of AVM	1 year	Transarterial embolization with NBCA is effective in treating intraorbital AVM
16	Coca et al. (2013) [22]	1	Not specified	42	Case Report	Madrid, Spain	Progressive proptosis, restriction of ocular motility, decreased visual acuity, chemosis	Orbital	20 x 15 x 15 mm	Compartment syndrome	CT, orbital angiography	Enhancing intraconal mass with arterial feeders from the pterygopalatine branch of the internal maxillary artery and ophthalmic artery	Manual carotid compression	High risk of embolization due to AVM location	Not applicable (manual compression)	None noted	Resolution of proptosis, improvement in visual acuity	1 year	Manual carotid compression is a viable non-invasive treatment option for AVMs with high embolization risks
17	Ayman (2014) [23]	1	Male (100%)	57 years	Case Report	Cairo, Egypt	Proptosis, hyperemic eye, swelling of eyelids, total ophthalmoplegia, pulsating sensation, headache, no light perception	Orbital	1.5 cm	None reported	CT, MRI, angiography	High-flow AVM fed by the ophthalmic artery, with numerous feeders from external carotid artery branches and draining into superior ophthalmic and facial veins	Embolization via direct venous approach using facial vein puncture	Preoperative embolization decision due to the lesion’s complex vascularity	Direct percutaneous venous puncture	None noted	Complete occlusion of AVM, significant relief in symptoms, no pulsation post-procedure	8 months	Direct venous embolization is an effective strategy for intraorbital AVMs with complex arterial supply
18	Patel et al. (2017) [24]	1	Female (100%)	15 years	Case Report	San Francisco, USA	No light perception in the right eye, gradually decreasing vision in the left eye	Retinal	N/A	Central retinal vein occlusion, perivascular sheathing, temporal nonperfusion	Widefield fluorescein angiography, OCT, MRI	Large AVM in the posterior pole of the right eye with extensive dilation and tortuosity; left eye appeared normal	Conservative management	Not applicable (observation recommended)	N/A	N/A	Stable condition with observation	Not specified	Isolated retinal AVMs are rare; conservative management is generally recommended unless complications arise
19	Sinha et al. (2017) [25]	1	Male (100%)	26 years	Case Report	Los Angeles, USA	Left-sided supraorbital mass, bluish non-tender mass with a palpable pulse	Orbital	4 cm	None reported	MRI, cerebral angiogram	AVM fed by the left superficial temporal, internal maxillary, and ophthalmic arteries	Endovascular embolization with NBCA glue followed by surgical resection	Progressive symptoms and cosmetic concerns	Embolization followed by excision of the lesion	Systemic urticarial reaction without cardiopulmonary compromise. Managed with antihistamines; symptoms improved	Both procedures were well tolerated and the patient was discharged home on the day of surgery	Not specified	Rare hypersensitivity reaction to NBCA glue; successful management with oral antihistamines
20	Tsui et al. (2019) [26]	1	Female (100%)	31 years	Case Report	New York, USA	Right-sided proptosis, pulsating mass palpable through the upper eyelid, scleral vessel engorgement	Orbital (superotemporal orbit, lacrimal gland region)	Not specified	None reported before treatment	CT angiography, Doppler imaging	High-flow AVM with major feeders from the distal branches of the ophthalmic artery and internal maxillary artery	Staged embolization with N-butyl cyanoacrylate (NBCA) glue followed by excision	Worsening symptoms after pregnancy (pain, headache)	Extended eyelid crease excision and lateral orbitotomy with bone window	None reported	Stable visual acuity, resolution of orbital deformity, planned future ptosis repair	11 months	Staged embolization and surgical excision provided successful management of orbital AVM, with good cosmetic and functional outcomes
21	Link et al. (2019) [27]	1	Female (100%)	31 years	Case Report	Kansas City, USA	Ocular infarction, light perception vision, proptosis, ophthalmoplegia, cherry red spot	Orbital	Not specified	Ocular infarction following treatment	Fluorescein angiography, indocyanine green angiography, MRI angiography	Decreased arteriolar filling, vascular leakage, decreased choroidal perfusion, full-thickness retinal edema	Ethanol sclerotherapy	Not applicable (sclerotherapy for AVM treatment)	N/A	Ocular infarction, vision loss	Significant visual impairment; managed post-sclerotherapy complications	Not specified	Highlighted the potential severe complications of ethanol sclerotherapy, including irreversible ocular infarction
22	Gandhi et al. (2020) [28]	1	Male (100%)	16 years	Case Report	New Delhi, India	Redness in the left eye, cosmetic concern due to progressive increase in size	Episcleral	10-11 mm vertically	None reported	MR angiography, contrast-enhanced MRI	Isolated episcleral AVM with feeder vessel from the anterior ciliary artery to the lateral rectus	Surgical excision	Cosmetic concern	Dissection of episcleral plane, excision of dilated vessels, conjunctival autograft from fellow-eye	None reported	No recurrence observed over 1-year follow-up	1 year	Isolated idiopathic episcleral AVM should be a diagnosis of exclusion, and management may include observation, embolization, or excision
23	Accou et al. (2020) [29]	1	Female (100%)	15 years	Case Report	Ghent, Belgium	Sudden-onset, painless visual loss in the right eye	Retinal	Not specified	Vitreous hemorrhage, retinal ischemia	Fluorescein angiography, MRI brain	Dilated, tortuous arteriovenous vessels extending from the optic disc, silver wiring of the enlarged vessels, macular microangiopathy, diffuse retinal nonperfusion	23-gauge pars plana vitrectomy, peripheral retinal photocoagulation	Persistent vitreous hemorrhage	Pars plana vitrectomy	None reported	Improved vision postoperatively	Not specified	Retinal arteriovenous malformations can remain stable but may cause complications like ischemia, warranting regular observation and timely intervention
24	Rosenblatt et al. (2021) [30]	1	Female (100%)	46 years	Case Report	Stanford, USA	Worsening left eye pain, eye redness, swelling, proptosis, restricted eye movement	Orbital	2.6 x 1.8 x 1.9 cm	Partial thrombosis, optic nerve compression	CT, MRI, CT angiography, digital subtraction angiography	High-flow AVM with multiple feeders from ciliary and lacrimal arteries, enlarged draining veins	Microsurgical resection with intraoperative digital subtraction angiography	Intractable pain, progression of proptosis	Anterolateral cranio-orbitotomy with microvascular clipping and angiographic guidance	None reported	Complete excision, resolution of symptoms, no recurrence at 6-year follow-up	6 years	Intraoperative digital subtraction angiography aids in precise microsurgical resection of orbital AVMs, minimizing risks and ensuring complete lesion removal
25	Dantas et al. (2021) [31]	1	Male (100%)	57 years	Case Report	Nova Lima, Minas Gerais, Brazil	Severe chemosis, proptosis, ophthalmoparesis, retro-orbital pain	Orbital	Not specified	Thrombosis, hemorrhage	CT, MRI, digital subtraction angiography (DSA)	Expansive intraorbital lesion with hemorrhage, fed by orbital branches of the right ophthalmic artery; thrombosis of the superior ophthalmic vein	Percutaneous embolization with coils and onyx through a transorbital puncture	Rapid progression of symptoms and AVM growth	Direct orbital puncture of the superior ophthalmic vein	Worsening chemosis postoperatively, managed with anticoagulants	Complete closure of the AVM, improvement in symptoms with residual mild proptosis at six months	6 months	First case of intraorbital AVM treated successfully with transorbital direct puncture, demonstrating a novel minimally invasive approach
26	Kamalden et al. (2008) [32]	1	Female (100%)	33 years	Case Report	Miami, FL, USA	Progressive proptosis, decreased visual acuity, afferent pupillary defect, compressive optic neuropathy	Orbital	2.5 x 4.5 cm	Vision loss due to ophthalmic artery occlusion	MRI, angiography, fluorescein angiography, indocyanine green angiography	High-flow AVM in the superotemporal intraconal space; post-embolization occlusion of the ophthalmic and central retinal arteries	Embolization with n-butyl cyanoacrylate (NBCA)	Progressive symptoms and compressive optic neuropathy	Not applicable; planned post-embolization excision was deferred	Complete vision loss, leading to enucleation	Enucleation due to painful blind eye; histopathology revealed emboli in the retinal and choroidal vasculature	3 months	Highlighted the severe risk of ophthalmic artery occlusion from NBCA embolization despite precautions, emphasizing the need for thorough patient counseling on potential complications
27	Li et al. (2024) [33]	1	Male (100%)	18 years	Case Report	Fuzhou, China	Progressive visual deterioration in the right eye	Optic nerve	Not specified	Visual field defects	MRI, CT angiography, d digital subtraction angiography (DSA)	Abnormal signal intensity within the optic nerve, vascular malformation involving the optic nerve	Conservative management	Not applicable; conservative management chosen to preserve visual function	Not applicable	None reported	No further deterioration in visual acuity or visual field observed during follow-up	Not specified	Optic nerve AVMs present significant diagnostic challenges and are often misdiagnosed. Conservative management can be effective in preserving visual function, though microsurgical intervention or embolization may be necessary in other cases

Clinical Manifestations and Diagnostic Modalities

Orbital AVMs presented with a wide range of symptoms related to the complexity and variability of the lesions. Chakrabortty et al. described a case with visual loss, exophthalmos, and chemosis due to an AVM involving the retrobulbar space and extraocular muscles [7]. Similarly, Gil-Salú et al. reported chemosis and an audible bruit unassociated with exophthalmos in a patient with an AVM affecting the extrabulbar fatty tissue [8]. These cases show that different clinical presentations depend on the precise anatomic location of the malformation.

Each diagnostic tool like CT, MRI, and angiography is uniquely suited for the evaluation of AVM characteristics in their own strengths and limitations. CT scans offer the best anatomical detail, enabling good identification of calcifications and bony involvement, which is very useful for surgical planning. However, sensitivity increases with CT in the assessment of soft tissues and vascular flow dynamics [7]. On the other hand, MRI provides better contrast of soft tissues, and delineation of the detailed structure of the AVM, including flow characteristics and extension into surrounding tissues. The major drawbacks of MRI are its sensitivity to motion artifacts and the requirement for longer acquisition times, which can be challenging for patients with acute symptoms [10].

In particular, angiography, mainly digital subtraction angiography (DSA), stands for the gold standard of vascular malformation investigation, as it provides real-time information on the dynamic passage of blood through an AVM, identifying feeders and draining veins with precision. It is an invasive technique and, therefore, involves a few risks, such as contrast-induced nephropathy and vascular injury [21]. The combination of these modalities allows for a complete evaluation that guides the clinician in decision-making and optimizes a treatment plan. Each modality provides important information that, taken together, enhances the overall understanding of AVM complexity and leads to a multidisciplinary treatment approach.

For example, Sato et al. employed DSA in the diagnosis of a high-flow AVM supplied by branches from the ophthalmic artery [21]. Pathak-Ray et al. illustrated dilated and tortuous retinal vessels in a patient with both a retinal and an orbital AVM using combined MRI and ultrasound [14]. Color Doppler sonography and fluorescein angiography had been performed in cases where determining the lesion's vascular structure and hemodynamic features was more complicated, as reported by Ho et al. [19].

Approaches to Treatment

The management of orbital AVMs in the reviewed series was based on size, location, flow dynamics, and clinical severity. The preferred surgical resection was indicated for cases with extreme symptoms such as proptosis, loss of vision, or when AVMs were situated in an area inaccessible to embolization. It was surgical approaches, such as fronto-orbital craniotomies or orbitotomies, as in the cases presented by Hieu et al. and Coca et al., that provided long-term resolution of proptosis and improved vision [11,22]. Generally speaking, surgery has been indicated in patients with symptomatic AVMs in whom conservative management or embolization has failed or is contraindicated due to the location of the lesion.

Surgical resection posed excessive risk near eloquent structures, especially in high-flow AVMs, making embolization the preferred option. For example, embolization methods described by Sato et al. were normally performed either as a single treatment or as an adjuvant to surgical intervention, depending on the complexity of the AVM [21]. It builds up the importance of cautious patient selection due to major complications, including occlusive events of key vessels leading to vision loss, as noted in the case by Kamalden et al. 2008 [32]. High-flow AVMs and those that drew a significant vascular supply from critical arteries probably benefited most from embolization, provided it was performed by an experienced team with risks clearly explained to the patient.

In cases where surgery or embolization may be riskier than any potential benefit, especially for slow-growing or asymptomatic AVMs, conservative management is the chosen treatment. For instance, Schatz et al. reported two cases in which intervention might have caused a high risk of loss of vision or other serious complications [9]. Thus, observations might be preferred for those presenting a stable lesion with few symptoms or for those unfit for surgery because of comorbidities. Conservative management was done with long-term stability in a case of slowly progressive proptosis, as demonstrated by Kaufman et al. [18].

In conclusion, surgical resection, embolization, or conservative management should be tailored to both the patient's clinical presentation and the AVM's characteristics, taking into consideration the general status of the patient. High-risk surgical candidates would be appropriate candidates for embolization, whereas conservative approaches are chosen for stable, low-risk cases or patients who cannot tolerate an invasive procedure.

Results and Complications

The outcomes varied depending on the treatment modality and the extent of the AVM. Surgical and embolization cases generally had better outcomes in terms of the resolution of proptosis, pain, or visual loss. Gil-Salú et al. described a fronto-orbital craniotomy that resulted in the complete removal of an intraorbital AVM with no recurrence during a four-year follow-up [8].

Trombly et al. documented a case of a pediatric patient with a high-flow AVM involving the eyelids and scalp, where serial embolization led to significant improvement in symptoms [17]. However, complications were not uncommon, particularly in cases of large or high-flow AVMs. In a study by Moin et al., a patient developed intraoperative hemorrhage during orbitotomy, ultimately leading to loss of light perception and the need for enucleation [13]. Despite the risks, embolization proved to be a valuable tool in managing AVMs, especially when used as an adjunct to surgery. Ho et al. described a patient with progressive visual loss and neovascular glaucoma who underwent gamma knife radiosurgery and transscleral cyclophotocoagulation, achieving partial symptom control [19]. However, in some cases, embolization alone was insufficient. Symptoms from incomplete embolization of a slow-flow AVM have persisted in the long term, with no significant improvement in visual outcomes [12].

The follow-up has varied from a few months in some series to more than six years. Long-term stability was observed in cases that were managed conservatively or after successful surgical or embolization procedures. Rosenblatt et al. supported the idea of intraoperative digital subtraction angiography for complete lesion removal and reported no recurrence of symptomatology or AVM in their series over a six-year follow-up [30].

Key Findings

This systematic review outlines the complexity of the management of orbital AVMs. The therapeutic options are tailored according to the nature of the AVM. Surgical resection is often effective but may entail major risks, especially in cases of high-flow or large AVMs. Embolization alone or combined with surgery is an effective option for treating high-risk lesions, though it still carries potential complications for visual loss and incomplete occlusion. Smaller or more stable AVMs may be amenable to conservative management; however, these should be kept under extremely close follow-up to monitor for progression or complications, especially in optic nerve or retinal involvement.

Discussion

Orbital AVMs are a direct, abnormal connection between arterial and venous systems, associated at times with increased hemodynamic stress and elevated central venous pressure (CVP). Intraoperative monitoring of CVP becomes an important measure, particularly during resection, because it presents real-time information about pressure dynamics in the venous system and, in general, on the success of surgical intervention. A significant decrease in CVP after resection indicates effective disruption of the abnormal vascular shunt, with the restoration of normal flow. The decrease in pressure acts indirectly to indicate the completeness of both resection and vascular correction, in addition to minimizing complications like hemorrhage. Intraoperative imaging, especially angiography, will help in the exact localization and ligation of the feeder vessels of the malformation to allow the complete removal of the lesion while preserving critical orbital structures.

Coupling these modern monitoring modalities with judicious surgical planning in orbital AVM resection may minimize complications such as venous congestion, residual malformation, and visual morbidity [34]. The results of this systematic review emphasize the diversity of symptomatology, modes of diagnosis, different treatment modalities, and clinical outcomes, hence indicating that an individualized multidisciplinary modality of treatment is essential in dealing with these lesions.

Indeed, the clinical manifestations of orbital AVMs ranged from mild cosmetic concerns to severe vision-threatening symptoms in the cases reviewed. Proptosis and visual impairment were largely accepted as common symptoms, just like the case reports by Chakrabortty et al. and Hieu et al. [7,11]. The location, size, and hemodynamic characteristics of the AVM will dictate a large part of the symptomatology. Symptoms of high-flow AVM had the following presentations that were rather more acute and aggressive: pain, chemosis, and rapid visual deterioration. For example, Sato et al. reported a case where severe pulsating exophthalmos and ocular pain due to a high-flow AVM resolved only after embolization [21].

Most orbital AVMs in the literature review relied on imaging studies as an important part of workup. Indeed, MRI, CT, and angiography were the most frequent imaging modalities used in the reviewed cases. Angiography was able to give critical information regarding vascular architecture and flow dynamics. Particularly for high-flow lesions, some advanced techniques include DSA and fluorescein angiography, which were very useful in the evaluation and in the planning of surgery. Kaufman et al. illustrated the value of MRI and angiography in the delineation of the entire extent of orbital involvement and vascular feeder patterns that guided appropriate treatment [18].

Management of AVM involving the orbit always heralds a peculiar challenge owing to the delicate anatomy of the orbit and the complicating rates of hemorrhage, loss of vision, and recurrence. Treatment strategies across the cases varied significantly; the most common interventions were surgical resection and embolization. Surgical resection is often the treatment of choice in patients with large or progressively symptomatic AVMs. For example, in a study by Hieu et al., a large intraorbital AVM was successfully treated with a fronto-orbital craniotomy coupled with CO₂ laser, with a result of full recovery of vision and complete resolution of proptosis [11]. Surgical risks are considerable; however, as in the case of Moin et al., in which calamitous intraoperative hemorrhage led to irreversible loss of vision beyond recovery and hence required enucleation [13]. These cases emphasize the need for preoperative planning and evaluation of the risks versus benefits of surgery.

Embolization has become an important adjunct or alternative treatment, particularly for AVMs characterized by high flow. Being able to devitalize vascular supply to the lesion prior to surgery can decrease intraoperative complications, such as hemorrhage. Sato et al. demonstrate that NBCA arterial embolization is indeed effective; complete occlusion of the AVM reduced symptoms [21]. However, embolization itself bears its risks. Shoji et al. reported that embolization has been associated with serious complications such as ophthalmic artery occlusion with subsequent permanent vision loss [32]. This case points to the need for careful patient selection and appropriate risk management in AVM embolization, especially in lesions that involve critical ocular structures.

When surgical or embolization methods were too risky or not indicated, conservatism was undertaken. Schatz et al. also reported on the follow-up of two cases of retinal AVM; one case remained stable after eight years, while another case progressed to neovascular glaucoma [9]. These findings would thus suggest that conservative management may be appropriate in a number of small or asymptomatic AVMs, although close monitoring is certainly warranted to provide early detection of an AVM that is showing signs of progression or complications.

The outcomes of the treatments for orbital AVM depended on the size, flow characteristics, and location of the malformation. Generally, in cases of complete resection or embolization, outcomes were favorable, associated with resolution of symptoms and minimal recurrences. Gil-Salu et al. reported long-term stability without recurrence in a patient with a large intraorbital AVM treated by fronto-orbital craniotomy, thus demonstrating the possibility of a surgical cure in selected cases [8]. However, complications were frequent, as would be expected, in particular in cases with high-flow AVMs or those presenting extensive involvement of critical structures. The most frequent complication reported was hemorrhage - intraoperative and postoperative - as reported by Moin et al. [13]. Similarly, the inability to achieve total resection of the AVM in a pediatric patient due to brisk intraoperative bleeding of the lesion was described by Trombly et al., thus resulting in progressive necrosis and deterioration of function [17]. Again, a multidisciplinary approach concerning the management of complex AVMs through neurosurgeons, interventional radiologists, and ophthalmologists aims to minimize complications and provide an optimal outcome.

Other major complications include visual loss, especially in AVMs involving the optic nerve or the retinal vasculature. Shoji et al. put forward a severe case of loss of vision following embolization due to occlusion of the ophthalmic artery as a reminder of the balance that needs to be considered while managing AVMs proximate to vital structures responsible for vision [32].

Most cases, despite these challenges, gained long-term stability after the intervention was successfully done, as was evidenced in a study by Rosenblatt et al., showing no recurrence or further complications after complete resection of a high-flow AVM for six years of follow-up [30].

Thus, the integrated approach using interventional radiology and surgery has become the most successful strategy in the management of these injuries. Preoperative endovascular embolization reduces flow through the malformation, thereby minimizing intraoperative hemorrhage and enhancing success rates for surgical resection. Even the combined approach can ensure a more controlled and safer excision of the AVM, reducing the risk of incomplete resection or recurrence.

Accurate diagnosis, often aided by sophisticated imaging studies such as angiography or MRI, is crucial in differentiating the AVM from other vascular malformations like capillary hemangiomas or arteriovenous fistulas. The early institution of a tailored, multidisciplinary treatment affords superior functional and cosmetic outcomes, reducing the long-term morbidity of these high-flow vascular anomalies [35].

In 2011, Van Went et al. described a case of spontaneous thrombosis of an orbital AVM leading to the diagnosis of HHT or Rendu-Osler-Weber disease [36]. HHT is a rare genetic disorder that manifests as abnormal vascular formations. An AVM most commonly affects the lungs and brain but involves the orbit infrequently. Management of orbital AVMs in the setting of HHT is challenging, given the high risk that embolization - very often used in brain and pulmonary AVMs - carries to occlude either the retinal artery or vein, leading to irreversible loss of vision. Thus, a conservative attitude was adopted in this case with steroid therapy and antiglaucomatous eye drops, with temporary improvement of ocular symptoms. However, the patient was suffering from central retinal vein thrombosis with permanent visual impairment in the course of a subsequent study. In the study, the authors explain "the difficult management of orbital AVMs in HHT suffers from a so-called balancing between the risk of spontaneous complications and that of interventional treatments, such as embolization." Given the rarity of such lesions and the high risk of complications, management indeed remains a challenge in dealing with AVMs of the orbit.

Recent advances in imaging and treatment methods may be expected to improve the outcome of patients with orbital vascular malformations. Digital angiography with subtraction techniques allows great detail of AVMs, thereby precisely delineating the vascular feeders and flow dynamics, which is of paramount importance in planning interventions like embolization. Novel embolization materials, such as N-butyl cyanoacrylate (NBCA), provide effective occlusion of abnormal vessels with minimal destruction of healthy surrounding tissues. All these emerging technologies, integrated with multidisciplinary approaches, may significantly enhance the efficacy of treatment and reduce complications [37].

The heterogeneity of the included studies in this systematic review limits this study to mainly single-case reports or small case series, making generalization very difficult. The small sample sizes reduce the ability to capture the full spectrum of clinical presentations and treatment outcomes. Publication bias can also be an issue since studies with successful outcomes have a higher likelihood of being published, thereby creating potential data skew. Because no randomized controlled trials were found, and because of the limited long-term follow-up, it is not possible to draw firm conclusions on the efficacy and safety of any treatments.

Further advances in imaging and interventional techniques, such as the use of intraoperative digital subtraction angiography, form promising tools that may further improve surgical precision and outcomes. There remains a great need for investigation of ways to optimize treatment strategies, especially in the case of high-flow lesions, for which embolization carries significant risks. Additionally, the elaboration of more subtle classification systems of the orbital AVMs may allow a better decision of treatment, especially in terms of the timing of intervention or the choice of appropriate modalities. Though the ISSVA classification provides an understanding of vascular anomalies in general, this concept of orbital AVMs does not fully address the special anatomic and functional aspects related to the orbit.

## Conclusions

Given their rarity and complex vascular anatomy, there is a great challenge in the diagnosis and management of orbital AVMs. The variability in clinical presentations, which ranged from mild proptosis to severe vision loss, further underlines the need to have treatment strategies that may best be described as individualized and multidisciplinary. This systematic review further denotes that advanced imaging modalities such as MRI, CT, and angiography are highly essential in the exact diagnosis of AVMs that would guide appropriate intervention. Whereas surgical resection and embolization comprise the cornerstone of the management strategy, both modalities have inherent risks, with higher risks in high-flow AVMs, while common complications include hemorrhage and loss of vision. Embolization has emerged as an important preoperative adjunct and helps decrease intraoperative complications; however, selection of patients is exceedingly critical to preclude adverse outcomes. Stable asymptomatic cases are amenable to conservative management, including regular follow-up and close monitoring to prevent disease advancement. This review serves only to emphasize the requirement for tailored approaches, which are derived from collaboration between surgical, interventional, and radiological disciplines for optimum outcomes. Further studies are required to outline the protocols for treatment, particularly those concerning high-flow lesions, and to present a subtle classification of the orbital AVMs. A better understanding of pathophysiology and treatment outcomes advances the possibility of improving the prognosis and quality of life in patients with such lesions.

## References

[1] Warrier S, Prabhakaran VC, Valenzuela A, Sullivan TJ, Davis G, Selva D (2008). Orbital arteriovenous malformations. Arch Ophthalmol.

[2] Colletti G, Biglioli F, Poli T (2019). Vascular malformations of the orbit (lymphatic, venous, arteriovenous): diagnosis, management and results. J Craniomaxillofac Surg.

[3] Tawfik HA, Dutton JJ (2022). Orbital vascular anomalies: a nomenclatorial, etiological, and nosologic conundrum. Ophthalmic Plast Reconstr Surg.

[4] Stacey AW, Gemmete JJ, Kahana A (2015). Management of orbital and periocular vascular anomalies. Ophthalmic Plast Reconstr Surg.

[5] Page MJ, McKenzie JE, Bossuyt PM (2021). The PRISMA 2020 statement: an updated guideline for reporting systematic reviews. Int J Surg.

[6] Gagnier JJ, Kienle G, Altman DG, Moher D, Sox H, Riley D (2013). The CARE Guidelines: consensus-based clinical case reporting guideline development. Glob Adv Health Med.

[7] Chakrabortty S, Nagashima T, Izawa I (1993). Intraorbital arteriovenous malformation: case report. Surg Neurol.

[8] Gil-Salú JL, González-Darder JM, Vera-Román JM (2004). Intraorbital arteriovenous malformation: case report. Skull Base.

[9] Schatz H, Chang LF, Ober RR, McDonald HR, Johnson RN (1993). Central retinal vein occlusion associated with retinal arteriovenous malformation. Ophthalmology.

[10] Schumacher M, Wakhloo AK (1994). An orbital arteriovenous malformation in a patient with origin of the ophthalmic artery from the basilar artery. AJNR Am J Neuroradiol.

[11] Hieu PD, Besson G, Roncin S, Nonent M (1997). Successful surgical treatment of intraorbital arteriovenous malformations: case report. Neurosurgery.

[12] Huna-Baron R, Setton A, Kupersmith MJ, Berenstein A (2000). Orbital arteriovenous malformation mimicking cavernous sinus dural arteriovenous malformation. Br J Ophthalmol.

[13] Moin M, Kersten RC, Bernardini F, Kulwin D, Biddinger PW, Ernst RJ, Khouri LM (2000). Spontaneous hemorrhage in an intraorbital arteriovenous malformation. Ophthalmology.

[14] Pathak-Ray V, Lawson J, Harris M (2001). Retinal and orbital arteriovenous malformations. Eye (Lond).

[15] Lee KY, Gazzard G, Tan DT (2003). Ciliary body arteriovenous malformation?. Eye (Lond).

[16] Shields JA, Streicher TF, Spirkova JH, Stubna M, Shields CL (2006). Arteriovenous malformation of the iris in 14 cases. Arch Ophthalmol.

[17] Trombly R, Sandberg DI, Wolfe SA, Ragheb J (2006). High-flow orbital arteriovenous malformation in a child: current management and options. J Craniofac Surg.

[18] Kaufman Y, Cole P, Dauser R, Hollier L Jr (2007). Intraorbital arteriovenous malformation: issues in surgical management. J Craniofac Surg.

[19] Ho TY, Wang AG, Lin MC, Yen MY (2010). Orbital arteriovenous malformation. Jpn J Ophthalmol.

[20] Rouvas A, Petrou P, Ladas I, Vergados I, Andipa ES, Papathanasiou M, Markomichelakis N (2011). Extensive unilateral visual field loss due to peripapillary arteriovenous malformation. Int Ophthalmol.

[21] Sato K, Matsumoto Y, Kondo R, Tominaga T (2011). Intraorbital arteriovenous malformation treated by transarterial embolization: technical case report. Neurosurgery.

[22] Coca J, Romero R, Sanchez-Orgaz M, Arbizu A, Frutos-Martinez R, Fernandez-Prieto A, Marin-Aguilera B (2013). Use of carotid compression in a patient with arteriovenous malformation. Orbit.

[23] Ahmed AZ (2014). Direct percutaneous venous approach in the treatment of intra-orbital arterio-venous malformation. Egypt J Radiol Nucl Med.

[24] Patel KH, Kalevar A, McDonald HR, Johnson RN (2017). Retinal arteriovenous malformation. Retin Cases Brief Rep.

[25] Sinha KR, Duckwiler G, Rootman DB (2017). Urticarial reaction following endovascular embolization of an orbital arteriovenous malformation (AVM) with N-butyl cyanoacrylate (NBCA) glue. Interv Neuroradiol.

[26] Tsui E, Dunbar KE, Kim ET, Patel P (2019). Staged embolization and excision of an arteriovenous malformation involving the eyelid and orbit. Orbit.

[27] Link T, Kam Y, Ajlan R (2019). Ocular infarction following ethanol sclerotherapy of an arteriovenous malformation. Am J Ophthalmol Case Rep.

[28] Gandhi A, Naik M, Mehta A (2020). Congenital isolated idiopathic episcleral arteriovenous malformation. Am J Ophthalmol Case Rep.

[29] Accou GP, Nerinckx F, Leroy BP, De Zaeytijd J (2020). Vitreous hemorrhage as presenting sign of retinal arteriovenous malformation. Case Rep Ophthalmol Med.

[30] Rosenblatt TR, Myung D, Fischbein NJ, Steinberg GK, Kossler AL (2021). Microsurgical resection of an orbital arteriovenous malformation with intraoperative digital subtraction angiography. Ophthalmic Plast Reconstr Surg.

[31] Dantas F, Carvalho TS, Firmino RU, Yung AA, Gomes RC, Mendes GA, Darwich RZ (2021). Percutaneous treatment of an intraorbital arteriovenous malformation using a transvenous approach: a case report. Interv Neuroradiol.

[32] Kamalden TA, Choo MM, Kadir K, Khaliddin N (2008). Arterial occlusion following embolization of an orbital arteriovenous malformation. A case report. Neuroradiol J.

[33] Li J, Lin F, Zhao M, Kang D, Lin Y, Wang D (2024). Uncommon optic nerve arteriovenous malformation: a case report and literature review. J Stroke Cerebrovasc Dis.

[34] Starks VS, Gilliland G, Hise J, Thacker I, Layton KF (2015). Effect of resection of an orbital arteriovenous malformation on central venous pressure. Proc (Bayl Univ Med Cent).

[35] Mukherjee B, Vijay V, Halbe S (2018). Combined approach to management of periocular arteriovenous malformation by interventional radiology and surgical excision. Indian J Ophthalmol.

[36] Van Went C, Ozanne A, Saliou G (2011). Spontaneous thrombosis of an orbital arteriovenous malformation revealing hereditary haemorrhagic telangiectasia (Rendu-Osler-Weber disease). A case report. Interv Neuroradiol.

[37] Shoaib KK, Mehmood A (2023). Orbital vascular malformations - clinical presentation and management strategies. Pak J Ophthalmol.

